# The T3SS Effector Protease NleC Is Active within *Citrobacter rodentium*

**DOI:** 10.3390/pathogens10050589

**Published:** 2021-05-12

**Authors:** Md Kamrul Hasan, Samir El Qaidi, Philip R. Hardwidge

**Affiliations:** College of Veterinary Medicine, Kansas State University, Manhattan, KS 66506, USA; kamrulbd@ksu.edu (M.K.H.); selqaidi1@yahoo.fr (S.E.Q.)

**Keywords:** T3SS effector, NleC, *Citrobacter rodentium*, p65, NF-κB

## Abstract

Whether type III secretion system (T3SS) effector proteins encoded by Gram-negative bacterial pathogens have intra-bacterial activities is an important and emerging area of investigation. Gram-negative bacteria interact with their mammalian hosts by using secretion systems to inject virulence proteins directly into infected host cells. Many of these injected protein effectors are enzymes that modify the structure and inhibit the function of mammalian proteins. The underlying dogma is that T3SS effectors are inactive until they are injected into host cells, where they then fold into their active conformations. We previously observed that the T3SS effectors NleB and SseK1 glycosylate *Citrobacter rodentium* and *Salmonella enterica* proteins, respectively, leading to enhanced resistance to environmental stress. Here, we sought to extend these studies to determine whether the T3SS effector protease NleC is also active within *C. rodentium*. To do this, we expressed the best-characterized mammalian substrate of NleC, the NF-κB p65 subunit in *C. rodentium* and monitored its proteolytic cleavage as a function of NleC activity. Intra-bacterial p65 cleavage was strictly dependent upon NleC. A p65 mutant lacking the known CE cleavage motif was resistant to NleC. Thus, we conclude that, in addition to NleB, NleC is also enzymatically active within *C. rodentium*.

## 1. Introduction

Gram-negative bacteria interact with host cells and inject virulence factors (effectors) through type III secretion system (T3SS) machineries [1]. Many *Escherichia coli* and *Citrobacter rodentium* T3SS effectors inhibit pro-inflammatory pathways, including NleB [2], NleH [3], EspL [4], NleD [5], and NleE [6].

NleC is a zinc metalloprotease T3SS effector that cleaves the NF-κB p65 subunit and thus inhibits p65 nuclear translocation. This dampens the host innate immune response, particularly the production of inflammatory cytokines [7,8,9,10,11]. NleC proteolytic activity is lost upon mutagenesis of the consensus zinc metalloprotease motif _183_HEIIH_187_ [11]. Although NleC is secreted through the T3SS machinery, it also has the capacity to behave as a short-trip toxin and be internalized independently of the T3SS [12]. Other substrates of NleC other than p65 have been described and include the Rel family member p50 [8] and the acetyltransferase p300 [13]. It is thought that NleC specifically targets the NF-κB pathway instead of the MAPK pathway since it shows no activity against STAT or ERK [7,9].

Although two distinct p65 recognition and cleavage sites were initially described [7,11,14], it is now established that the cleavage site is C38/E39 [15,16]. NleC recognition of its substrates is also influenced by residues that are distant from the cleavage site [15,17].

The traditional view of T3SS effector secretion is that the effectors are inactive until injected into host cells, where they then fold into their active conformations and interact with host targets [18]. However, our recent work has challenged this dogma. We discovered that the T3SS effector NleB is active not only within the host, but also within *C. rodentium*. NleB activity with *C. rodentium* results in glycosylation of the glutathione synthetase (GshB) to enhance GshB activity and cause increased production of glutathione, leading to protection from oxidative stress [19]. We also observed that the NleB ortholog SseK1 is active within *Salmonella enterica*, where it enhances methylglyoxal detoxification [20]. As these data suggested that T3SS effectors may be active within the bacterium, here we desired to investigate the potential intra-bacterial effector of an effector with a different enzymatic activity. To do so, we investigated whether this phenomenon also applied to the *C. rodentium* zinc metalloprotease protease NleC.

## 2. Results

**Figure 1:**
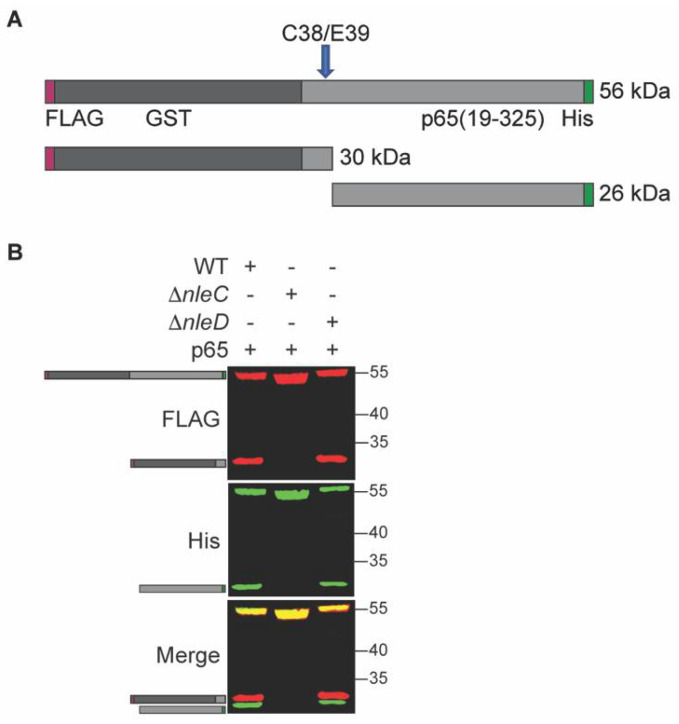
NleC cleaves p65 in *C. rodentium*. (**A**). Schematic. p65 (residues 19-325) was cloned and expressed as a recombinant fusion to N-terminal FLAG (red)- and GST (grey)-tags and a C-terminal His-tag (green). The cleavage site is indicated with an arrow; the N-terminal and C-terminal cleavage products with epitope tags and their expected sizes are also shown. (**B**). p65 cleavage is dependent upon NleC. p65 was expressed in WT, Δ*nleC*, or Δ*nleD C. rodentium*. Protein lysates were analyzed using Western blotting. FLAG, His, and merged blot images are shown. Cartoons indicate the expected full-length and cleavage products as depicted in panel A.

To investigate NleC activity inside *C. rodentium*, we cloned its known eukaryotic substrate p65 into a prokaryotic expression vector. Since the NleC cleavage site of p65 is near the N-terminus, we added a glutathione S-transferase (GST) tag to the N-terminus of p65 to increase the size of cleavage products. We also added a FLAG tag to the N-terminus to further aid detection of the cleavage product, as well as a C-terminal His-tag to aid with purification. Thus, the final recombinant p65 construct has a His-tag at the C-terminus and GST- and FLAG tags at the N-terminus. If cleavage by NleC occurs, the recombinant p65 protein is expected to be cleaved into two fragments: a 30 kDa N-terminal FLAG-tagged fragment and 26 kDa C-terminal His-tagged fragment (Figure 1A).

To investigate the NleC mediated cleavage of p65, p65 expression plasmids introduced into wild-type (WT) *C. rodentium*, or into deletion strains lacking NleC or NleD (Δ*nleC*), and (Δ*nleD*) strains. We observed that p65 was cleaved into the two expected fragments of appropriate molecular weights in the WT strain, but no such cleavage products were seen in the Δ*nleC* strain. Additionally, the same cleavage products were also seen in the Δ*nleD* strain, a strain lacking the T3SS effector NleD, a zinc metalloprotease that cleaves JNK [5] (Figure 1B). Thus, p65 cleavage within *C. rodentium* appears to be dependent upon NleC.

**Figure 2:**
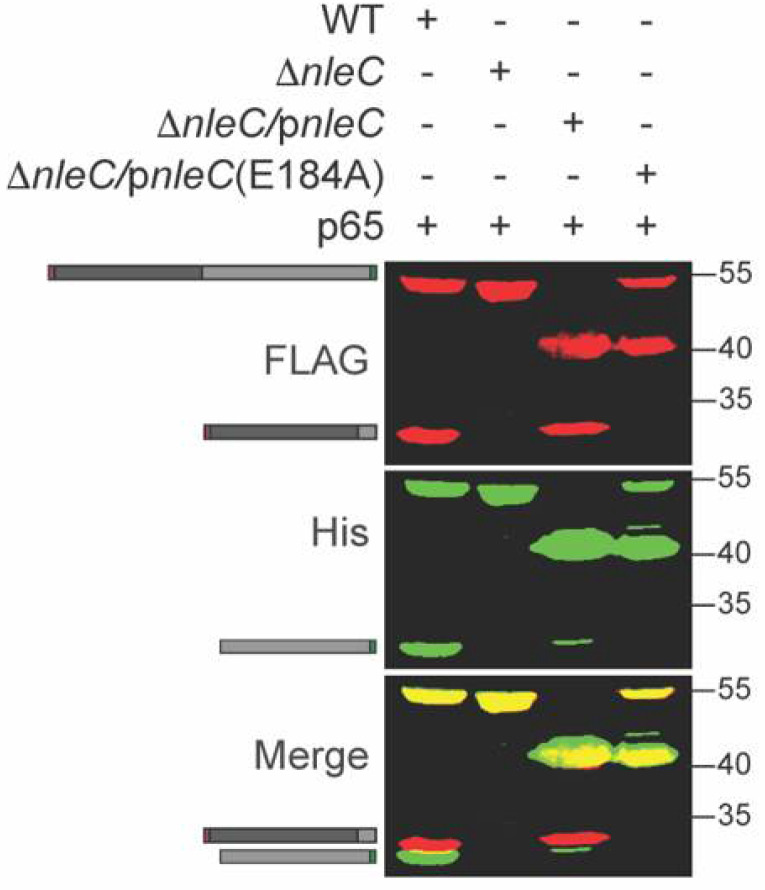
Inactive NleC does not cleave p65. p65 was expressed in WT, Δ*nleC*, or Δ*nleC* complemented with either WT NleC or NleC E184A *C. rodentium*. Experiments were conducted as described in Figure 1 panel B. The 40 kDa protein band is the NleC protein produced via plasmid complementation.

NleC enzymatic activity is significantly reduced in the host cell when the critical NleC residue E184 around the Zn^2+^ binding pocket is mutated to A184 [7]. To further evaluate p65 cleavage specificity and its dependence upon NleC activity, we co-transformed Δ*nleC C. rodentium* with a p65 expression plasmid and either WT NleC or NleC E184A complementation plasmids. The recombinant p65 was cleaved by WT NleC but not by NleC E184A (Figure 2).

**Figure 3:**
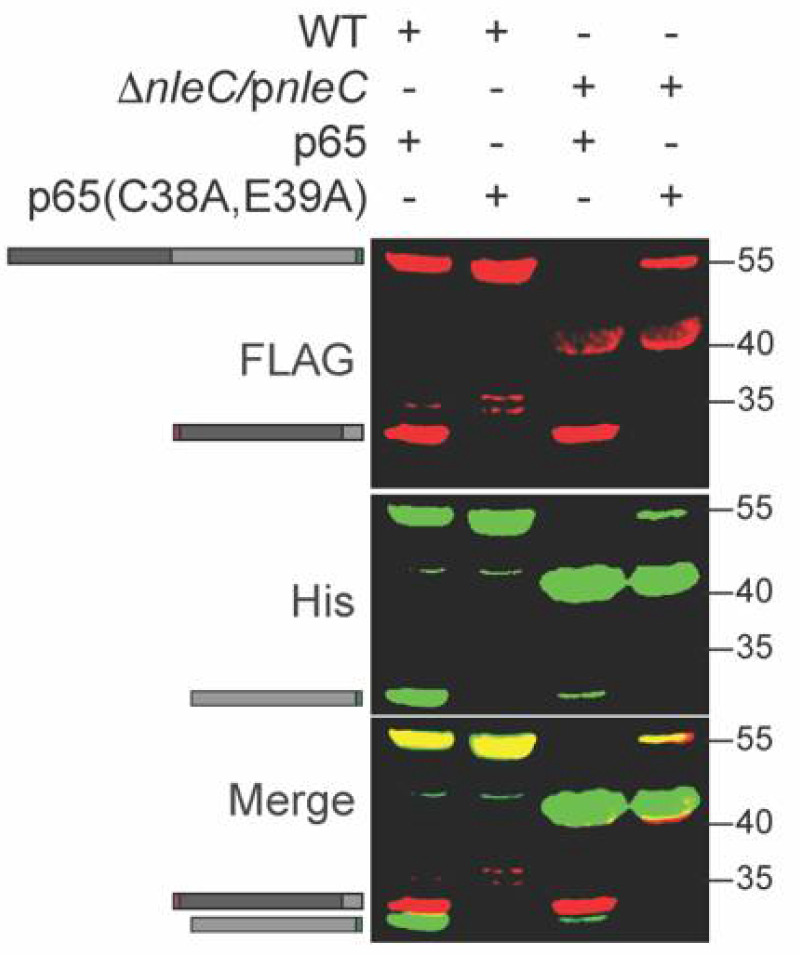
NleC does not cleave the p65 (C38A/E39A) mutant. WT and mutant p65 were expressed in WT *C. rodentium*. Experiments were conducted as described in Figure 1 panel B.

p65 is cleaved by NleC between C38 and E39 [15]. To determine whether this cleavage specificity was retained within *C. rodentium*, we mutated the p65 expression plasmid to change C38/E39 to alanine residues. We expressed this plasmid in WT *C. rodentium* and observed that the p65 C38A, E39A mutant was no longer cleaved by NleC (Figure 3). In addition, we also purified the His-tagged (C-terminal) fragment of the cleavage product (Figure 3, lane 1) and performed Edman degradation analyses. The five N-terminal amino acids we identified corresponded to the expected sequence (EGRSA) of the C-terminal cleavage product (data not shown). Thus, we concluded that NleC is active within *C. rodentium.*

**Figure 4:**
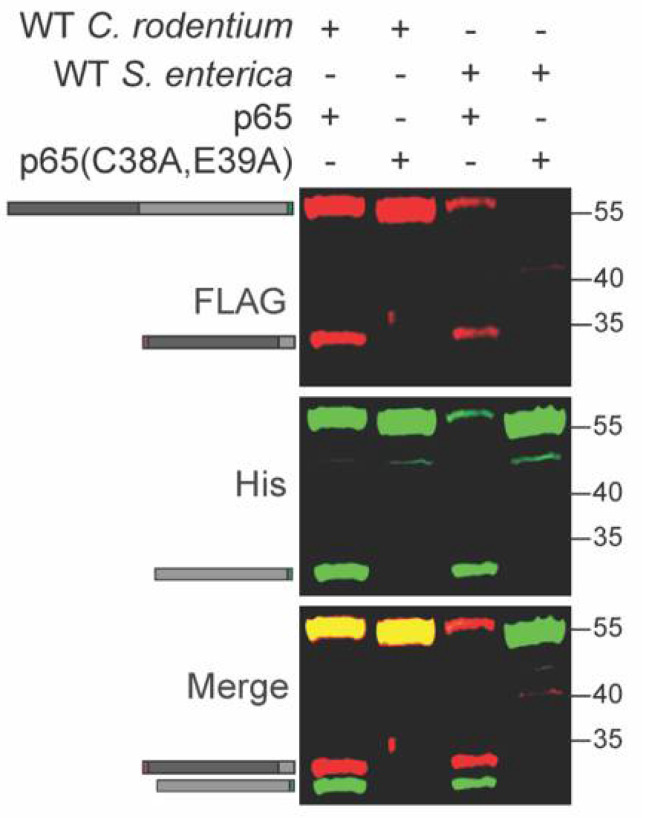
p65 cleavage occurs within *S. enterica*. WT and mutant p65 were expressed in WT *S. enterica*. Experiments were conducted as described in Figure 1 panel B.

*Salmonella enterica* also induces p65 cleavage in host cells [21,22]. We tested whether p65 is cleaved inside *S. enterica* in a similar fashion to *C. rodentium*. We expressed the recombinant WT and C38A/E39A p65 constructs in *S. enterica* and observed cleavage of only the WT p65 construct, similarly to what was observed for *C. rodentium* (Figure 4 and Table S1). *S. enterica* harbors at least three distinct T3SS effectors, GogA, PipA, and GtgA, that cleave p65 inside host cells [22]. At least one of these effectors may be active within *S. enterica*.

## 3. Discussion

NleC is a zinc metalloprotease that cleaves the NF-κB p65 subunit and dampens the immune response of the host cell. Despite the traditional view of T3SS effectors being active only after their translocation into the host, here we demonstrated that the *C. rodentium* T3SS effector NleC is active inside the bacterium. The implication of this novel finding is far-reaching. As demonstrated by our previous findings of the *C. rodentium* T3SS effector NleB glycosylating the bacterial substrate GshB and therefore contributing to the increased survival of *C. rodentium* under environmental stress [19], as well as *S. enterica* SseK1 glycosylating multiple bacterial substrates involved in methylglyoxal (MGO) detoxification [20], NleC may have its own endogenous bacterial targets. Compared with Locus of Enterocyte Effacement (LEE)-encoded effectors, non-LEE-encoded effectors such as NleB and NleC are potentially more recent acquisitions to the pathogens and their roles as effectors are still evolving [23]. This indicates that non-LEE effectors potentially have a dual role in modulating both pathogen and host cellular pathways. Therefore, NleC activity in *C. rodentium* could play a significant role in modulating bacterial regulation of virulence, response to environmental stress, and immune evasion.

The traditional model of T3SS effector translocation is that the effectors are chaperoned within the bacterium until they are secreted. Indeed, chaperones for multiple effectors have been identified and characterized [24,25,26,27,28]. To our knowledge, no chaperone for NleC has been identified, indicating that NleC might not be chaperoned before its secretion, potentially explaining its activity inside the bacteria. Similarly, no chaperone has been identified for either NleB1 [19] or SseK1.

The potential intra-bacterial targets of NleC are currently unknown, and this absence of knowledge potentially limits the biological impact of our findings. However, some insight could be obtained from bioinformatics analyses. For example, we performed an initial bioinformatics-based search for bacterial NleC substrates by using the known p65 cleavage site as a query. One potential hit is GrlR, a regulator of LEE expression [29] that harbors both the conserved NleC cleavage site and a DNA-binding domain. In theory, NleC could cleave GrlR and thus relieve the repression of LEE expression, which in turn could increase the expression and/or secretion of NleC and other effectors. Another study found that NleC recognizes and bind its target protein by DNA mimicry, which indicates that some other targets of NleC may be proteins with nucleic acid binding capacity [16]. NleC may thus indirectly regulate transcription and affect bacterial responses to environmental changes. Proteomic techniques such as Terminal Amine Isotopic labeling of Substrates (TAILS) [30] might also reasonably be employed to identify proteins whose abundance is dependent upon NleC activity.

## 4. Materials and Methods

### 4.1. Strains and Molecular Cloning

Plasmids were constructed in pET15 and pET28 backgrounds by using ABC cloning [31]. p65 and NleC point mutants were created by using site-directed mutagenesis. *C. rodentium* Δ*nleC* and Δ*nleD* strains were constructed by using lambda red recombination [32] with pKD3 and pKD119 plasmids. A recombination cassette harboring a selectable marker flanked by ~50 bp of complementary sequences from the 5′ and 3′ ends of the gene to be deleted was created by using PCR with pKD3 as a template. The *nleC* recombination cassette was amplified by using forward (5′-GCTCCCATTGCTCCTAATCGTGCTGAAAATGCCTATGCGGATGAAATCTAACAATGCGCT) and reverse (5′-GCCAATCCAGGAAAATCATGTTGCTGGATAAATCCGTATCGGTCGAGGTGGCCCGGCTCC) primers. The *nleD* recombination cassette was amplified by using forward (5′-GCTCAATGTCAGATACAGATATCGAGTCTCTTGTAAAAGCAAGCCACTGGAGCACCTCAA) and reverse (5′-CGGGTAGGAGGTTCTACGGGGCATCCCAATCTCTTCGTGGACGGGGAGAGCCTGAGCAAA) primers. Plasmids were introduced into *C. rodentium* and *S. enterica* strains by using electroporation.

### 4.2. Western Blotting

Strains were grown in LB media with appropriate antibiotics (kanamycin and/or carbenicillin) for 4 h at 37 °C and then induced with 0.5 mM IPTG with growth for an additional 4 h at 30 °C. Cell lysates were derived from 1 mL of bacterial cultures via centrifugation and lysed in 100 µL SDS loading buffer by boiling for 5 min. Samples (10 µL) were used in Western blot assays with anti-His (1:1000 dilution; H1029, Sigma-Aldrich, St. Louis, SL, USA) and anti-FLAG (1:5000 dilution; F7425, Millipore, Burlington, USA) primary antibodies. IRDye 800CW goat-anti-mouse IgG (926-32210, LI-COR) and IRDye 680RD goat-anti-rabbit IgG (926-68071, LI-COR) secondary antibodies were used at 1:10,000 dilutions.

### 4.3. Edman Degradation

The C-terminal p65 cleavage product generated from WT *C. rodentium* was purified using standard His-tagged protein purification methods and processed according to the sample preparation guidelines for Edman degradation by the Protein Facility of the Iowa State University Office of Biotechnology.

## Data Availability

Data are contained within the article and supplemental file.

## Supplementary Materials

The following are available online at https://www.mdpi.com/article/10.3390/pathogens10050589/s1, Table S1: Edman degradation analysis of p65 C-terminal cleavage product.
