# Supplementary material for: The T3SS Effector Protease NleC Is Active within Citrobacter rodentium
Source: Pathogens. 2021 May 12;10(5):589. doi: 10.3390/pathogens10050589 (PMC8151275; doi:10.3390/pathogens10050589)
Supplement: Supplementary file 1 [file pathogens-10-00589-s001.zip › pathogens-1204732-supplementary.pdf]

Supplementary Table. Edman degradation analysis of p65 C-terminal cleavage product.

Cycle #	PTH-amino acid	Yield (pmol) Replicate 1	Yield (pmol) Replicate 2
1	Glu (E)	2.5	6.2
2	Gly (G)	15.7	19.7
3	Arg (R)	19.4	49.5
4	Ser (S)	5.9	3.4
5	Ala (A)	3.5	6.5
